# RMBase: a resource for decoding the landscape of RNA modifications from high-throughput sequencing data

**DOI:** 10.1093/nar/gkv1036

**Published:** 2015-10-12

**Authors:** Wen-Ju Sun, Jun-Hao Li, Shun Liu, Jie Wu, Hui Zhou, Liang-Hu Qu, Jian-Hua Yang

**Affiliations:** 1 Key Laboratory of Gene Engineering of the Ministry of Education, Sun Yat-sen University, Guangzhou 510275, P. R. China; 2 State Key Laboratory for Biocontrol, Sun Yat-sen University, Guangzhou 510275, P. R. China

## Abstract

Although more than 100 different types of RNA modifications have been characterized across all living organisms, surprisingly little is known about the modified positions and their functions. Recently, various high-throughput modification sequencing methods have been developed to identify diverse post-transcriptional modifications of RNA molecules. In this study, we developed a novel resource, RMBase (RNA Modification Base, http://mirlab.sysu.edu.cn/rmbase/), to decode the genome-wide landscape of RNA modifications identified from high-throughput modification data generated by 18 independent studies. The current release of RMBase includes ∼9500 pseudouridine (Ψ) modifications generated from Pseudo-seq and CeU-seq sequencing data, ∼1000 5-methylcytosines (m^5^C) predicted from Aza-IP data, ∼124 200 N6-Methyladenosine (m^6^A) modifications discovered from m^6^A-seq and ∼1210 2′-O-methylations (2′-O-Me) identified from RiboMeth-seq data and public resources. Moreover, RMBase provides a comprehensive listing of other experimentally supported types of RNA modifications by integrating various resources. It provides web interfaces to show thousands of relationships between RNA modification sites and microRNA target sites. It can also be used to illustrate the disease-related SNPs residing in the modification sites/regions. RMBase provides a genome browser and a web-based modTool to query, annotate and visualize various RNA modifications. This database will help expand our understanding of potential functions of RNA modifications.

## INTRODUCTION

Post-transcriptional modification of RNA molecules occurs in all living organisms, and is one of the most evolutionarily conserved properties of RNAs (1–5). It can affect the activity, localization as well as stability of RNAs, and has been linked with human diseases (1–5).

Although more than 100 types of RNA modifications have been described so far, most of them were thought to be abundant in tRNAs, rRNAs and snRNAs, but rare in mRNAs and in regulatory non-coding RNAs (ncRNAs). To determine the transcriptome-wide landscape of RNA modifications, recently many studies have developed high-throughput modification sequencing methods to identify diverse post-transcriptional modifications of RNA molecules (1–5). Application of these methods has identified various modifications (e.g. pseudouridine, m^6^A, m^5^C, 2′-O-Me) within coding and non-coding sequences at single nucleotide or very high resolution (6–17). With the increasing amount of modification sequencing data available, there is a great need to integrate these large-scale data sets to explore the prevalence, mechanism and function of various modifications.

Many novel functional roles of RNA modifications have been revealed by functional experiments in recent years. For example, m^6^A has been predicted to affect protein translation and localization (1–5) or mRNA stability (18) and stem cell pluripotency (19,20). Pseudouridylation of nonsense codons suppresses translation termination both *in vitro* and *in vivo*, suggesting that RNA modification may provide a new way to expand the genetic code (21). Importantly, many modification enzymes are dysregulated and genetically mutated in many disease types (1). For example, genetic mutations in pseudouridine synthases cause mitochondrial myopathy, sideroblastic anemia (MLASA) (22) and dyskeratosis congenital (23). However, the relationships between genetic variants identified from genome-wide association studies (GWAS) and modification sites identified by above-mentioned various high-throughput methods were yet unexplored.

**Figure 1.**
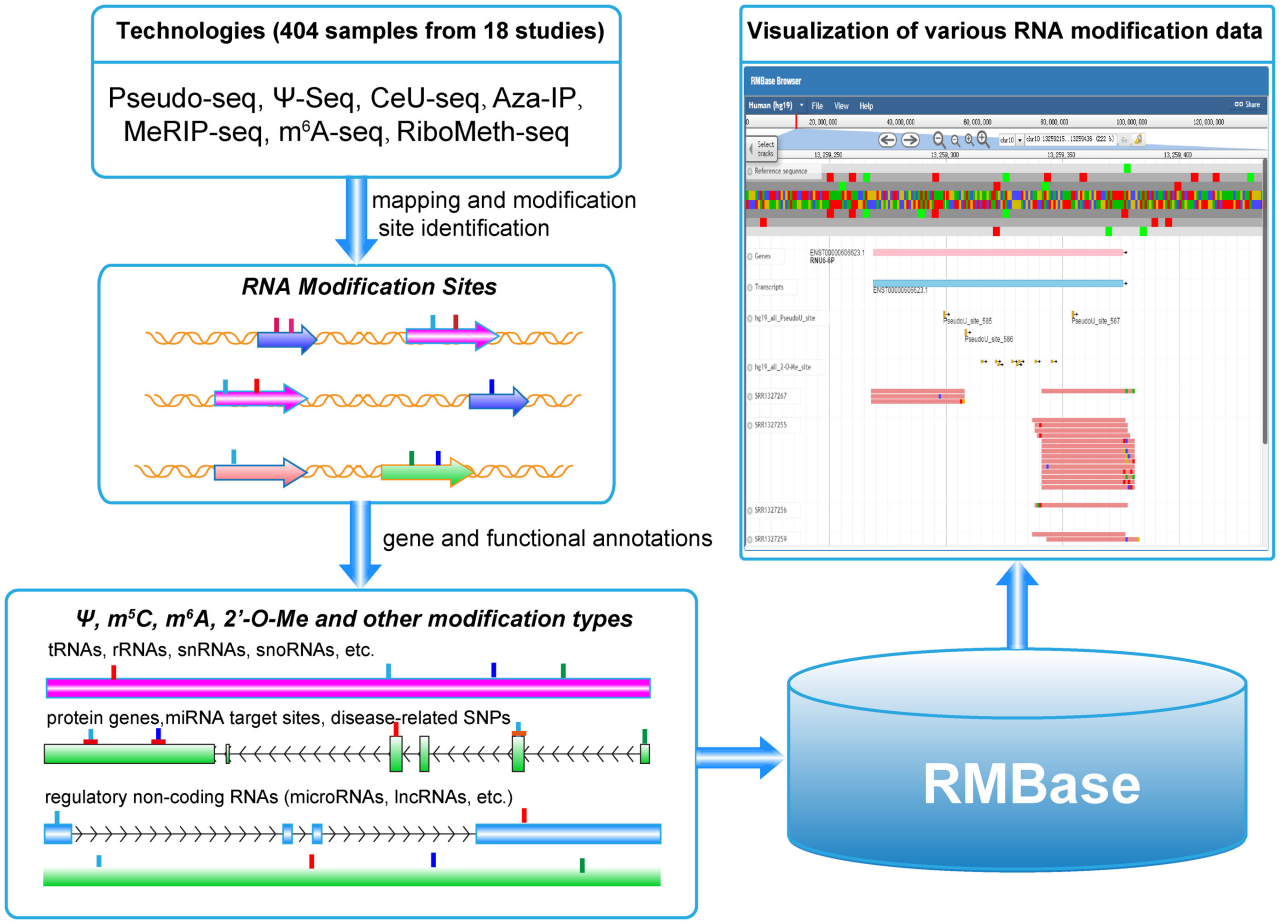
System overview of RMBase core framework. We integrated a large set of RNA modification sites generated by 18 independent studies to profile the comprehensive genome-wide modification landscape of more than 100 types of RNA modifications. Integrative analysis of RNA modification sites has shown extensive post-transcriptional modification of RNA. Our combined analysis of RNA modification data with GWAS and miRNA target data identified thousands of miRNA targets and disease-related SNPs resided in the modification sites. High-throughput modification sequencing data were mapped to genomes and displayed in genome browser. All results generated by RBMBase are deposited in MySQL relational databases and displayed in the visual browser and web page.

In this study, we developed RMBase to facilitate the annotation, visualization, analysis and discovery of RNA modification sites from large-scale modification sequencing data (Figure 1). In RMBase, we performed a large-scale integration of public RNA modification sites generated by high-throughput sequencing technology, and provided the RNA epigenetic map for various cell types that are presently available (Table 1). RMBase provides web interfaces to show the relationships between miRNA targets and RNA modifications. Furthermore, by integrating GWAS data into database, RMBase can be used to illustrate the clinically relevant RNA modification sites. As the integration of more than 100 types of RNA modifications, it is expected to help the researchers to investigate the potential functions and mechanisms of RNA modifications.

**Table 1.** The data statistics in RMBase

species	Ψ	m^5^C	m^6^A	2′-O-Me	Other types
Human	4128	680	94 895	901	617
Mouse	3247	97	28 002	66	497
Yeast	2122	211	1306	242	2014

Statistics indicating the numbers of each modification type for the three organisms, including human, mouse, yeast. Ψ is pseudouridine modification, m^5^C is 5-methylcytosine methylation, m^6^A is N6-Methyladenosine methylation and 2′-O-Me is 2′-O-methylation, rare modification types are integrated into as ‘other types’.

## MATERIALS AND METHODS

### Integration of public high-throughput modification sequencing data sets

High-throughput Pseudo-seq, CeU-seq, Aza-IP, m^6^A-seq, MeRIP-Seq and RiboMeth-seq data were retrieved from the Gene Expression Omnibus (GEO) and the supplementary data of the original references (6–17). Barcodes or 3′-adapters of raw modification sequencing data were clipped using the FASTX-toolkit software (version 0.0.13). All unique reads without adapters in each sample were mapped to genomes using Bowtie 1.1.2 (24).The mapping reads were converted into BAM format and displayed in genome browser. Known modifications for rRNAs, snRNAs and tRNAs were extracted from snoRNABase (25), MODOMICS (26), Yeast snoRNA Database (27) as well as other literature sources (28–30), and then were mapped to genome using Bowtie program (24) to determine the genomic coordinates and construct the genome-wide landscape of RNA modifications.

### Annotation of modification sites

All gene annotations were downloaded from UCSC bioinformatics websites (31) and Ensembl (32). Human (UCSC hg19), mouse (UCSC mm10, NCBI Build 38), Yeast (sacCer3) genome sequences were also downloaded from UCSC bioinformatics websites (31). All modification sites were annotated using above-mentioned annotation data sets. Modification sites were classified into the following gene types: tRNAs, rRNAs, Mt-tRNAs, scRNAs, snRNAs, snoRNAs, miRNAs, lincRNAs, misc_RNAs, protein-coding genes, processed_transcripts, pseudogenes, etc. and genomic regions which include CDS, 3′-UTR, 5′-UTR, intron, exon and intergenic.

### Identification and annotation of m^6^A modification sites

To obtain high resolution m^6^A modification sites, we predicted exact m^6^A posi­tions from MeRIP-Seq or m^6^A-seq peaks by searching for consensus DRACH (where D denotes A, G or U, R denotes A or G and H denotes A, C or U) motifs as described by previous study (17,33). All these exact m^6^A positions were annotated as the above-mentioned descriptions.

### Identification of disease-related SNPs in modification sites

As described in our previous study (34), disease/phenotype associated SNPs were curated from published GWAS data provided by the NHGRI GWAS Catalog (35), Johnson and O'Donnell (36), dbGAP (37) and GAD (38). Additional SNPs in linkage disequilibrium (LD) with reported disease-related loci were selected with the criteria requiring an r^2^ value over 0.5 in at least one of the four populations (CEU, CHB, JPT and YRI) genotype data of the HapMap project (release 28) (39). For each SNP, rs ID was lifted to dbSNP bulid 141 based on the ‘RsMergeArch.bcp’ and ‘SNPHistory.bcp’ table from dbSNP, and genomic coordinates were lifted to the hg19 assembly using the UCSC LiftOver tool. All these disease-related SNPs or LD SNPs were intersected with the modification regions, extended by an additional 10 nt in both the 5′- and 3′-directions for each modification site. Modification regions were defined according to the binding length of modification synthases (1,4), such as Fibrillarin (FBL, the methyltransferase) bind to complementary regions with at least 10 nt (40).

### Association analysis of miRNA targets with RNA modification sites

All miRNA-target interactions for human and mouse were downloaded from our starBase platform (41,42). All miRNA target sites were intersected with RNA modification sites to identify modifications that may influence miRNA-target interaction.

### Database and web interface implementation

All data sets were processed and stored in a MySQL Database Management System. The database query and user interface were developed using PHP and JavaScript. The query result table is based on jQueryUI and DataTables, which is a highly flexible tool for sorting and filtering the search result.

### RMBase genome browser

We constructed RMBase Genome Browser to provide an integrated view of reference sequences, modification sequencing data, aligned sequencing reads, RNA modification sites, protein-coding genes, ncRNA genes and transcripts. RMBase Browser is built on JBrowse (43) which is a fast, smooth scrolling and zooming genome browser.

## DATABASE CONTENT AND WEB INTERFACE

### The genome-wide landscape of various RNA modification types

We integrated 139 025 RNA modification sites generated by 18 independent studies to profile the genome-wide modification landscape of more than 100 types of RNA modifications (Table 1). To provide more useful information, we generated extensive annotations and analyses for all RNA modification sites. Therefore, RMBase can be used to show the modification sites of distinct modification types varied from several to thousands, and the genomic context distributions of modification sites for different types distinguished from each other.

### Annotating the association between RNA modifications and miRNA target sites

To help users investigate the association between RNA modifications and miRNA target sites, we collected all CLIP-Seq experimentally supported miRNA target sites from starBase database (41) and associated these data with all RNA modification sites from RMBase. RMBase allows users to retrieve all the RNA modification sites located within miRNA binding sites reported so far.

### Predicting GWAS-associated modification sites

Although GWAS have revealed a significant number of genetic variants related to diseases or phenotypes, a considerable portion of these identified loci remain not been functionally explained to date (44). To help users explore whether some modifications may be the real causation for diseases or phenotypes, we collected a total of 87 677 unique disease-related SNPs from four public GWAS data source. In addition, we also performed LD analysis to extract SNPs that had high LD relationship with disease-related SNPs using a threshold of r^2^ > 0.5 in at least one population from the HapMap CEU, CHB, JPT and YRI genotype data, which yielded a total of 895 968 disease-related or LD SNPs (34). By comparing the genomic coordinates of SNPs with all modification sites in human, RMBase can be used to illustrate the disease-related SNPs which are mapped to modification sites.

### The web-based exploration of different types of RNA modification sites

We provided five web interfaces (Pseudouridine/Ψ, m^6^A, m^5^C, 2-O-Me and otherType) which may be used to display RNA modification sites from various modification types. For each type of the RNA modification, users can select species in the query page. In the result page, the basic information of modification sites was displayed in a data table which includes 10 distinct fields to describe the details of modification sites. For each interface, the numbers of RNA modification sites are indicated in bottom-left corner of table. The user can also click on the title of the table to sort RNA modification sites according to various features, such as chromosome, genome positions, the number of supporting experiments, modId, the gene names or the gene types. User can input the keyword in search box to filter the results. The users can click on a modId within the table to launch a detailed page that provides further information about the RNA modification site in question. The detailed information for a modification site includes a description of the modification site, the list of supporting experiments and sequence that was extended by an additional 20 nt in both the 5′- and 3′-directions for the modification site. The ‘PubMed ID’ section enabled the retrieval of the primary articles yielding the modification data. Click the ID link to visit the NCBI PUBMED website.

The interface for modSNP and modMirTar was also provided and organized similarly to the above-mentioned interface, as well as disease-related SNP and miRNA-target interaction information. Users can explore their relationships between modification sites and SNP or miRNA target sites by similar ways.

### Visualization of various modification sequencing data using the RMBase genome browser

**Figure 2.**
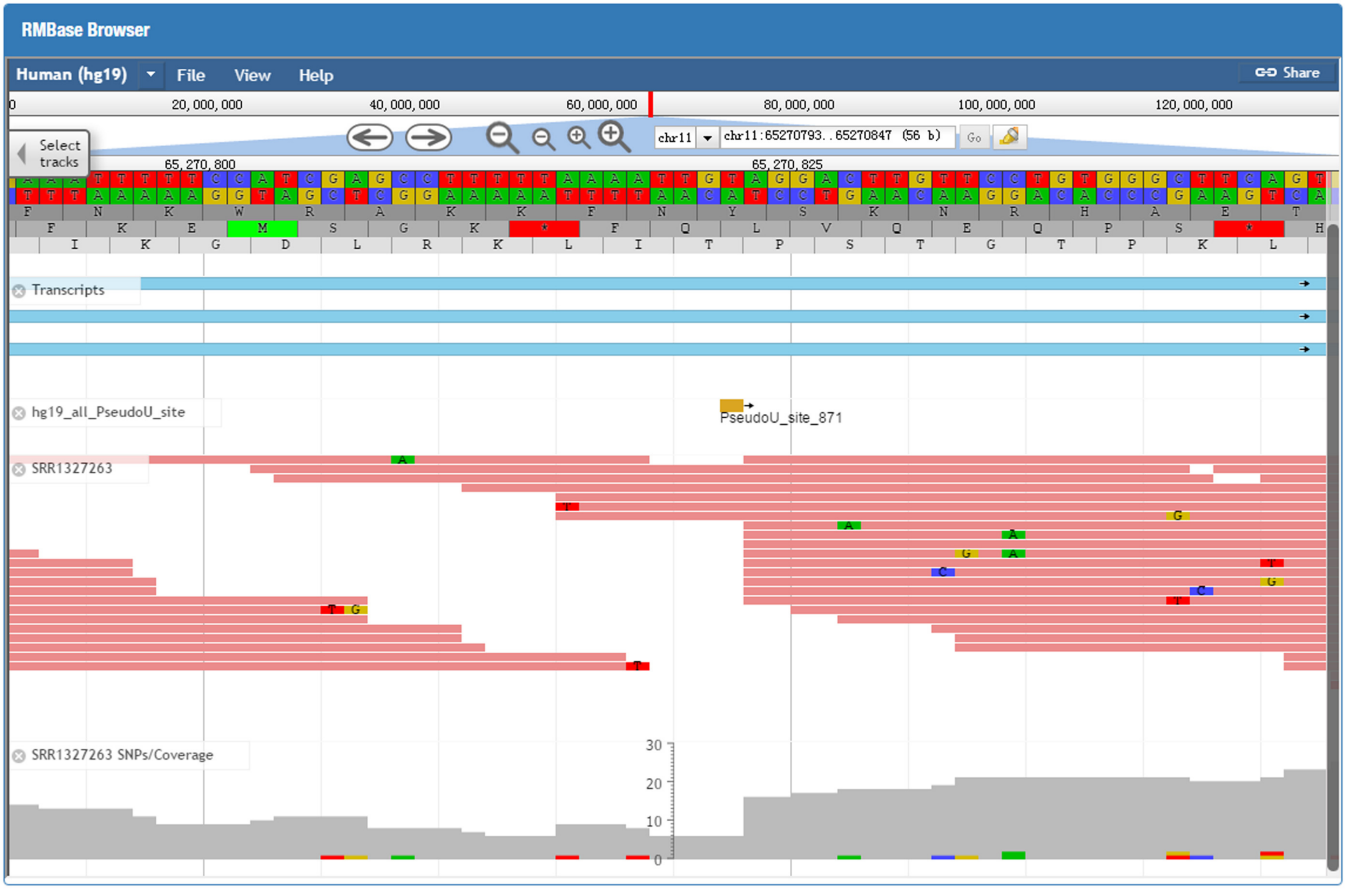
Illustrative screen shots from the RMBase genome browser. RMBase genome browser provides an integrated view of modification sequencing data, aligned sequencing reads, RNA modification sites, protein-coding genes, ncRNA genes.

To facilitate visualization of the various modification sequencing data sets and exploration of RNA modification sites, we provide RMBase genome browser that is built on JBrowse (43) (Figure 2). In the query page of the browser, users can input one interested genomic region or gene name in the ‘search term’ and select corresponding genome assembly to gain an integrated view of various genomic features. Information on RNA modification sites, aligned reads generated by modification sequencing methods, as well as gene annotations from Ensembl were provided. Figure 2 illustrated the visualization of genomic context for ‘PseudoU_site_871’ modification site located within MALAT1 lncRNA using RMBase Browser. Users can click the ‘+’ or ‘−’ button at the top to shrink or extend on the center of the annotation tracks window. Users can open the track select panel by clicking ‘Select Tracks’ button located in the upper-left corner and choose different types of modification data sets derived from various cell lines or treatments. To explore RNA modification sites on a particular gene, users can type its gene symbol in the position textbox and then click the ‘GO’ button to update the display image to determine what modification sites are located within the gene.

### Associating other data with modification sites using web-based modTool server

We provide the web-based modTool, which offers a simple and user-friendly interface to annotate modification sites in genomic regions uploaded by user. The user is required to select an intended organism and then upload genomic regions in the browser extensible data (BED) format. After the user has completed the data submission, a typical iteration of the modTool program may require several seconds or minutes to finish. The output of this program mainly consisted of a data table that included 10 distinct fields to describe the details of hits. The results include the query name, modification positions on genomes, modification type, the number of supporting experiments or studies, gene name, gene type (e.g. protein-coding or ncRNA) and regions (CDS, 3′ UTR, exon, 5′ UTR, intron, intergenic) on genes. Users can reorder any columns in the result table. Thus, it is convenient for data view and comparison in the user-defined vision style. Moreover, the keyword search was supported to scale down the results. Only 200 entries of hit information are displayed in the table, and users can obtain all results in text format by clicking on the ‘export’ button.

## DISCUSSION AND CONCLUSIONS

By integrating a large set of RNA modification sites derived from all available high-throughput modification sequencing methods (Pseudo-seq, CeU-seq, Aza-IP, MeRIP-Seq, m^6^A-seq, RiboMeth-seq) and public resources, RMBase reveals extensive post-transcriptional modification of RNA in mammalian and yeast.

In comparison with the other databases related to RNA modifications, including MODOMICS (26), RNAMDB (45) and MeT-DB (46) which collected modification sites identified by traditionally experimental methods or contain one modification type only, the advances of our RMBase database are as follows: (i) RMBase provides the annotation and analysis of various public modification sequencing data generated by Pseudo-seq, CeU-seq, Aza-IP, m^6^A-seq and RiboMeth-seq, which are the newest high-throughput technology for the transcriptome-wide identification of RNA modification sites in both animals and plants. (ii) RMBase provides the genome-wide landscape of pseudouridine (Ψ), m^5^C and 2′-O-Me modifications. (iii) RMBase provides genomic coordinates of all modification sites. This will facilitate computational or experimental biologists to correlate their results with all modification sites deposited in RMBase. (iv) RMBase allows combined analysis of RNA modification data and GWAS data, which identify hundreds of disease-related SNPs resided in the modification sites. These results will help to reveal the real causations and mechanisms for diseases or phenotypes identified from GWAS studies. (v) RMBase also illustrates relationships between RNA modification sites and miRNA target sites. (vi) In RMBase, we provided RMBase genome browser to provide a quick overview of a particular region in the genome and for visually correlating various types of features (Figure 2). This browser may provide an integrated view of modification sequencing data, RNA modification sites, protein-coding genes and ncRNA genes (Figure 2). (vii) RMBase provides the comprehensive annotation of various types of RNA modifications (Figure 1) and a new web-based tool, modTool, to annotate modification sites in genomic regions uploaded by user. (viii) RMBase provides a variety of interfaces and graphic visualizations to facilitate analysis of the massive and heterogeneous modification data in normal tissues and cancer cells.

Overall, RMBase allows an integrative analysis of various high-throughput modification data that provide insights into the epigenetic regulation of the transcriptome. As genome-wide high-throughput sequencing data for RNA modifications become more and more available, RMBase will help researchers further investigate these data and discover potential functional roles of RNA modifications hidden in these data.

## FUTURE DIRECTIONS

With the development of new high-throughput modification sequencing method, there will be more and more single nucleotide resolution modification sequencing data. We have built an automatic pipeline which is run in our high-performance computer servers to automatically annotate, analyze and merge all high-throughput modification data sets, and then import these data into our local MySQL database. We will continually maintain and update the database every two months or whenever new high-throughput modification data sets are released in public databases. RMBase will continue to expand the storage space and improve the computer server performance for storing and analyzing these new data, and we will develop or integrate new tools to decode the landscape of RNA modifications.

## AVAILABILITY

RMBase is freely available at http://mirlab.sysu.edu.cn/rmbase/. The RMBase data files can be downloaded and used in accordance with the GNU Public License and the license of primary data sources.

## References

[1] Li S., Mason C.E. (2014). The pivotal regulatory landscape of RNA modifications. Annu. Rev. Genomics Hum. Genet..

[2] Lee M., Kim B., Kim V.N. (2014). Emerging roles of RNA modification: m(6)A and U-tail. Cell.

[3] Song C.X., Yi C., He C. (2012). Mapping recently identified nucleotide variants in the genome and transcriptome. Nat. Biotechnol..

[4] Meyer K.D., Jaffrey S.R. (2014). The dynamic epitranscriptome: N6-methyladenosine and gene expression control. Nat. Rev. Mol. Cell Biol..

[5] Kirchner S., Ignatova Z. (2015). Emerging roles of tRNA in adaptive translation, signalling dynamics and disease. Nat. Rev. Genet..

[6] Carlile T.M., Rojas-Duran M.F., Zinshteyn B., Shin H., Bartoli K.M., Gilbert W.V. (2014). Pseudouridine profiling reveals regulated mRNA pseudouridylation in yeast and human cells. Nature.

[7] Schwartz S., Bernstein D.A., Mumbach M.R., Jovanovic M., Herbst R.H., Leon-Ricardo B.X., Engreitz J.M., Guttman M., Satija R., Lander E.S. (2014). Transcriptome-wide mapping reveals widespread dynamic-regulated pseudouridylation of ncRNA and mRNA. Cell.

[8] Chen K., Lu Z., Wang X., Fu Y., Luo G.Z., Liu N., Han D., Dominissini D., Dai Q., Pan T. (2015). High-Resolution N(6) -Methyladenosine (m(6) A) Map Using Photo-Crosslinking-Assisted m(6) A Sequencing. Angew. Chem..

[9] Khoddami V., Cairns B.R. (2013). Identification of direct targets and modified bases of RNA cytosine methyltransferases. Nat. Biotechnol..

[10] Birkedal U., Christensen-Dalsgaard M., Krogh N., Sabarinathan R., Gorodkin J., Nielsen H. (2015). Profiling of ribose methylations in RNA by high-throughput sequencing. Angew. Chem..

[11] Liu J., Yue Y., Han D., Wang X., Fu Y., Zhang L., Jia G., Yu M., Lu Z., Deng X. (2014). A METTL3-METTL14 complex mediates mammalian nuclear RNA N6-adenosine methylation. Nature Chem. Biol..

[12] Dominissini D., Moshitch-Moshkovitz S., Schwartz S., Salmon-Divon M., Ungar L., Osenberg S., Cesarkas K., Jacob-Hirsch J., Amariglio N., Kupiec M. (2012). Topology of the human and mouse m6A RNA methylomes revealed by m6A-seq. Nature.

[13] Meyer K.D., Saletore Y., Zumbo P., Elemento O., Mason C.E., Jaffrey S.R. (2012). Comprehensive analysis of mRNA methylation reveals enrichment in 3′ UTRs and near stop codons. Cell.

[14] Alarcon C.R., Lee H., Goodarzi H., Halberg N., Tavazoie S.F. (2015). N6-methyladenosine marks primary microRNAs for processing. Nature.

[15] Schwartz S., Agarwala S.D., Mumbach M.R., Jovanovic M., Mertins P., Shishkin A., Tabach Y., Mikkelsen T.S., Satija R., Ruvkun G. (2013). High-resolution mapping reveals a conserved, widespread, dynamic mRNA methylation program in yeast meiosis. Cell.

[16] Li X., Zhu P., Ma S., Song J., Bai J., Sun F., Yi C. (2015). Chemical pulldown reveals dynamic pseudouridylation of the mammalian transcriptome. Nature Chem. Biol..

[17] Linder B., Grozhik A.V., Olarerin-George A.O., Meydan C., Mason C.E., Jaffrey S.R. (2015). Single-nucleotide-resolution mapping of m6A and m6Am throughout the transcriptome. Nat. Methods.

[18] Wang X., Lu Z., Gomez A., Hon G.C., Yue Y., Han D., Fu Y., Parisien M., Dai Q., Jia G. (2014). N6-methyladenosine-dependent regulation of messenger RNA stability. Nature.

[19] Geula S., Moshitch-Moshkovitz S., Dominissini D., Mansour A.A., Kol N., Salmon-Divon M., Hershkovitz V., Peer E., Mor N., Manor Y.S. (2015). Stem cells. m6A mRNA methylation facilitates resolution of naive pluripotency toward differentiation. Science.

[20] Chen T., Hao Y.J., Zhang Y., Li M.M., Wang M., Han W., Wu Y., Lv Y., Hao J., Wang L. (2015). m(6)A RNA Methylation Is Regulated by MicroRNAs and Promotes Reprogramming to Pluripotency. Cell Stem Cell.

[21] Karijolich J., Yu Y.T. (2011). Converting nonsense codons into sense codons by targeted pseudouridylation. Nature.

[22] Bykhovskaya Y., Casas K., Mengesha E., Inbal A., Fischel-Ghodsian N. (2004). Missense mutation in pseudouridine synthase 1 (PUS1) causes mitochondrial myopathy and sideroblastic anemia (MLASA). Am. J. Hum. Genet..

[23] Heiss N.S., Knight S.W., Vulliamy T.J., Klauck S.M., Wiemann S., Mason P.J., Poustka A., Dokal I. (1998). X-linked dyskeratosis congenita is caused by mutations in a highly conserved gene with putative nucleolar functions. Nat. Genet..

[24] Langmead B., Trapnell C., Pop M., Salzberg S.L. (2009). Ultrafast and memory-efficient alignment of short DNA sequences to the human genome. Genome Biol..

[25] Lestrade L., Weber M.J. (2006). snoRNA-LBME-db, a comprehensive database of human H/ACA and C/D box snoRNAs. Nucleic Acids Res..

[26] Machnicka M.A., Milanowska K., Osman Oglou O., Purta E., Kurkowska M., Olchowik A., Januszewski W., Kalinowski S., Dunin-Horkawicz S., Rother K.M. (2013). MODOMICS: a database of RNA modification pathways–2013 update. Nucleic Acids Res..

[27] Piekna-Przybylska D., Decatur W.A., Fournier M.J. (2007). New bioinformatic tools for analysis of nucleotide modifications in eukaryotic rRNA. RNA.

[28] Yang J.H., Zhang X.C., Huang Z.P., Zhou H., Huang M.B., Zhang S., Chen Y.Q., Qu L.H. (2006). snoSeeker: an advanced computational package for screening of guide and orphan snoRNA genes in the human genome. Nucleic Acids Res..

[29] Maden B.E. (1990). The numerous modified nucleotides in eukaryotic ribosomal RNA. Prog. Nucleic Acid Res. Mol. Biol..

[30] Ofengand J., Bakin A. (1997). Mapping to nucleotide resolution of pseudouridine residues in large subunit ribosomal RNAs from representative eukaryotes, prokaryotes, archaebacteria, mitochondria and chloroplasts. J. Mol. Biol..

[31] Rosenbloom K.R., Armstrong J., Barber G.P., Casper J., Clawson H., Diekhans M., Dreszer T.R., Fujita P.A., Guruvadoo L., Haeussler M. (2015). The UCSC Genome Browser database: 2015 update. Nucleic Acids Res..

[32] Cunningham F., Amode M.R., Barrell D., Beal K., Billis K., Brent S., Carvalho-Silva D., Clapham P., Coates G., Fitzgerald S. (2015). Ensembl 2015. Nucleic Acids Res..

[33] Schwartz S., Mumbach M.R., Jovanovic M., Wang T., Maciag K., Bushkin G.G., Mertins P., Ter-Ovanesyan D., Habib N., Cacchiarelli D. (2014). Perturbation of m6A writers reveals two distinct classes of mRNA methylation at internal and 5′ sites. Cell Rep..

[34] Li J.H., Liu S., Zheng L.L., Wu J., Sun W.J., Wang Z.L., Zhou H., Qu L.H., Yang J.H. (2014). Discovery of Protein-lncRNA Interactions by Integrating Large-Scale CLIP-Seq and RNA-Seq Datasets. Frontiers Bioeng. Biotechnol..

[35] Welter D., MacArthur J., Morales J., Burdett T., Hall P., Junkins H., Klemm A., Flicek P., Manolio T., Hindorff L. (2014). The NHGRI GWAS Catalog, a curated resource of SNP-trait associations. Nucleic Acids Res..

[36] Johnson A.D., O'Donnell C.J. (2009). An open access database of genome-wide association results. BMC Med. Genet..

[37] Tryka K.A., Hao L., Sturcke A., Jin Y., Wang Z.Y., Ziyabari L., Lee M., Popova N., Sharopova N., Kimura M. (2014). NCBI's Database of Genotypes and Phenotypes: dbGaP. Nucleic Acids Res..

[38] Becker K.G., Barnes K.C., Bright T.J., Wang S.A. (2004). The genetic association database. Nat. Genet..

[39] Altshuler D.M., Gibbs R.A., Peltonen L., Altshuler D.M., Gibbs R.A., Peltonen L., Dermitzakis E., Schaffner S.F., Yu F., International HapMap, C. (2010). Integrating common and rare genetic variation in diverse human populations. Nature.

[40] Bachellerie J.P., Cavaille J., Huttenhofer A. (2002). The expanding snoRNA world. Biochimie.

[41] Li J.H., Liu S., Zhou H., Qu L.H., Yang J.H. (2014). starBase v2.0: decoding miRNA-ceRNA, miRNA-ncRNA and protein-RNA interaction networks from large-scale CLIP-Seq data. Nucleic Acids Res..

[42] Yang J.H., Li J.H., Shao P., Zhou H., Chen Y.Q., Qu L.H. (2011). starBase: a database for exploring microRNA-mRNA interaction maps from Argonaute CLIP-Seq and Degradome-Seq data. Nucleic Acids Res..

[43] Skinner M.E., Uzilov A.V., Stein L.D., Mungall C.J., Holmes I.H. (2009). JBrowse: a next-generation genome browser. Genome Res..

[44] Hindorff L.A., Sethupathy P., Junkins H.A., Ramos E.M., Mehta J.P., Collins F.S., Manolio T.A. (2009). Potential etiologic and functional implications of genome-wide association loci for human diseases and traits. Proc. Natl. Acad. Sci. U.S.A..

[45] Cantara W.A., Crain P.F., Rozenski J., McCloskey J.A., Harris K.A., Zhang X., Vendeix F.A., Fabris D., Agris P.F. (2011). The RNA Modification Database, RNAMDB: 2011 update. Nucleic Acids Res..

[46] Liu H., Flores M.A., Meng J., Zhang L., Zhao X., Rao M.K., Chen Y., Huang Y. (2015). MeT-DB: a database of transcriptome methylation in mammalian cells. Nucleic Acids Res..

